# Generating Explanations for Conceptual Validation of Graph Neural Networks: An Investigation of Symbolic Predicates Learned on Relevance-Ranked Sub-Graphs

**DOI:** 10.1007/s13218-022-00781-7

**Published:** 2022-11-07

**Authors:** Bettina Finzel, Anna Saranti, Alessa Angerschmid, David Tafler, Bastian Pfeifer, Andreas Holzinger

**Affiliations:** 1 University of Bamberg, Bamberg, Germany; 2 University of Natural Resources and Life Sciences, Vienna, Austria; 3 Medical University, Graz, Austria; 4 xAI-Lab, Alberta Machine Intelligence Institute, Edmonton, AB Canada

**Keywords:** Graph neural networks (GNN), Explainable AI (xAI), Inductive logic programming (ILP), Symbolic AI, Kandinsky pattern (KP)

## Abstract

Graph Neural Networks (GNN) show good performance in relational data classification. However, their contribution to concept learning and the validation of their output from an application domain’s and user’s perspective have not been thoroughly studied. We argue that combining symbolic learning methods, such as Inductive Logic Programming (ILP), with statistical machine learning methods, especially GNNs, is an essential forward-looking step to perform powerful and validatable relational concept learning. In this contribution, we introduce a benchmark for the conceptual validation of GNN classification outputs. It consists of the symbolic representations of symmetric and non-symmetric figures that are taken from a well-known Kandinsky Pattern data set. We further provide a novel validation framework that can be used to generate comprehensible explanations with ILP on top of the relevance output of GNN explainers and human-expected relevance for concepts learned by GNNs. Our experiments conducted on our benchmark data set demonstrate that it is possible to extract symbolic concepts from the most relevant explanations that are representative of what a GNN has learned. Our findings open up a variety of avenues for future research on validatable explanations for GNNs.

## Introduction

**Fig. 1:**
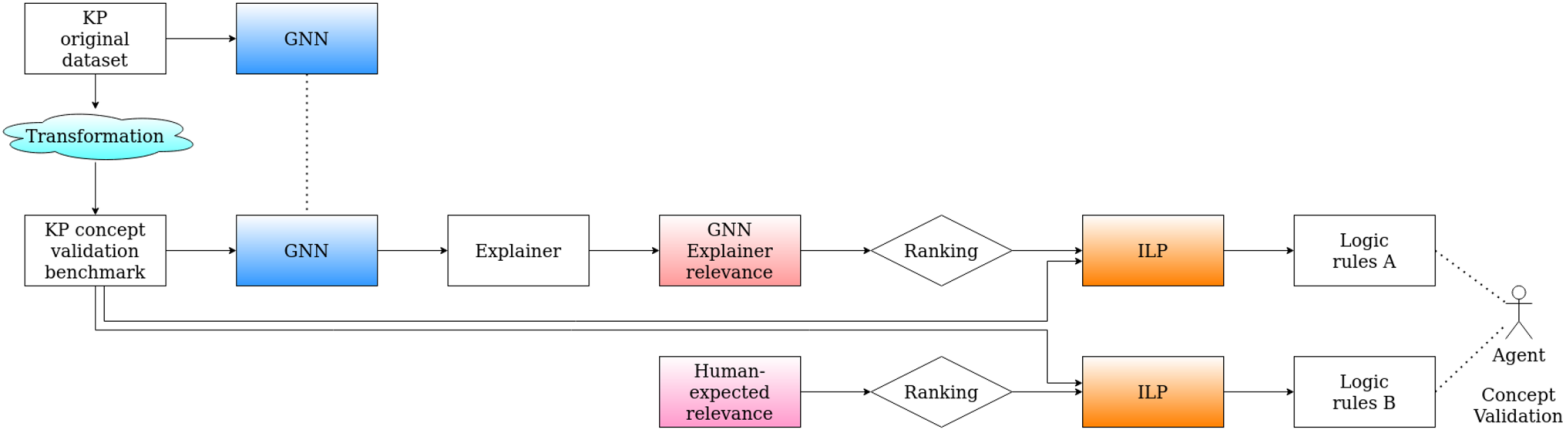
Overview of the operations in our approach. The Kandinsky Pattern (KP) original data set can be used individually to train a GNN. The same GNN (no retraining, freezed weights) is used to make predictions for the KP concept validation benchmark. Several state-of-the-art explainers (analyzed in Sect. 3.2) can be used to interpret the decision-making process of the GNN and compute relevance values for the important components of the input graphs (comprising nodes, edges, walks and sub-graphs). The graphs are ranked by their total relevance, divided into two classes according to a threshold, and comprise the input to ILP. ILP then produces a set of logic rules that serve as comprehensible explanations. Independently thereof, human-defined relevance that represents domain knowledge expectations, passes through a similar set of operations, producing another set of rules. The comparison of the two rule sets concerning generalization capabilities is the ultimate goal of this research work, and it can be performed by either a human or a computer agent

Machine learning models like neural networks became popular, e.g., for the classification of large amounts of data. However, maintaining transparency for human decision-makers remains an open issue due to the black-box character of such models [1]. Especially in the area of graph classification, where data has a complex structure, it is of great importance to gain insights into the reasons behind a classification outcome. Graphs consist of nodes and edges to represent, for example, manifold connections and relationships between entities like people, molecules, or locations [2]. The goal of graph classification is to find structures in a graph that are representative of a class (e.g., the molecular composition of a substance to distinguish carcinogenic and harmless chemicals [3]). This can be achieved, for instance, by generalizing on the similarity of attribute values, sub-graphs, on the size and the density of a graph and by considering distances between nodes in a network [4, 5].

Graph classification research has already introduced many suitable mathematical frameworks to find similarity and dissimilarity in graphs for distinguishing different classes as efficiently and reliably as possible, e.g., kernel-based and graph embedding methods [6–8], as well as logic-based approaches [8]. The high performance of Graph Neural Networks (GNNs), for example, in applications of biology and medicine makes them a promising approach to automated classification and knowledge discovery in large and complex data sets [3, 6, 9–11]. However, although using GNNs mostly eliminates the need for feature selection and the pre-definition of discriminating mathematical functions to fit different data distributions, it is still difficult or even impossible for a human to understand why a GNN has reached a classification decision and whether it used features for its generalization process that are relevant from an application domain and user’s perspective.

Approaches are needed that can be used to validate whether a GNN has learned concepts that can be described by humans. This is crucial for gaining an understanding of whether a GNN fulfills its purpose.

Methods for explaining GNNs are currently mainly based on identifying the most relevant input features w.r.t. the predictive outcome [12–14], e.g., by using generative techniques [15], decomposition by means of backpropagation [16], perturbations of input [17], approximation of decision boundaries with surrogate models [18, 19] or gradient-based methods [20]. Current xAI methods for GNNs are capable of extracting and highlighting the relevance for nodes, edges, features, paths, or even sub-graphs, depending on the particular approach. This is an important property because, as mentioned earlier, graphs are usually composed of complex, highly relational structures. The existing GNN explainers can thus help in detecting bias and overfitting in models [21] and their application can lead to actionable insights [22].

However, although the existing methods for explaining GNNs are based on techniques that have been found useful in the past, in part, in experiments and studies with humans [23, 24], there is still a lack of frameworks for evaluating the produced explanations for GNNs and relational data. Specifically, there is a need for methods to evaluate the output of a GNN w.r.t its conceptual validity. A concept can be represented by a human-understandable expression in contrast to raw features [25]. It can describe some property or relation, e.g., spatial relationships between cells extracted from medical images. A concept may further be characterized by sub-concepts. In analogy, a graph may be decomposed into sub-graphs, where some sub-graphs may represent relevant sub-concepts. This means that the human-understandable expression can be encoded as a sub-graph with its nodes’ and edges’ attributes.

There already exists work that evaluates GNNs w.r.t. learning concepts [26–31]. However, these works do not evaluate existing relevance-based GNN explainers regarding the fit between the relevance they compute and the features that represent important sub-concepts from an application domain’s and user’s perspective. Particularly, we identified three properties that limit the explanatory capabilities of current methods and that should be addressed by new evaluation frameworks.

First, the explanatory power of relevance is limited if it remains unknown whether a GNN has found sub-concepts to be relevant that are important from the perspective of the application domain or the user [32]. Therefore, a human should be involved in the evaluation of explanatory power. Second, the meaning of individual relevance values can be multifaceted and may depend on the context or classification problem [33]. Therefore, conceptual knowledge can help to assign explicit meaning to relevance. Last but not least, current explanatory approaches do not produce natural language statements or language-like outputs [34]. Visualizations may not be expressive enough, especially for complex, relational data [35–37]. Language accordingly facilitates conceptual validation complementary to visualizations and should therefore be incorporated into GNN evaluation frameworks.

In the last years, symbolic approaches, such as Inductive Logic Programming (ILP) regained their popularity due to their relational concept learning capabilities and their expressiveness as well as comprehensibility of nearly natural language expressions [38–41]. Further, symbolic AI promises to open a path to understandable descriptions for explaining, validating and improving the decisions of neural networks [35, 37, 42, 43]. Especially ILP supports learning from a small amount of data, and it shows promising results in generalizing from the output of neural network explainers, e.g., for explaining and validating the classification of relational concepts in images [35, 37]. Furthermore, ILP is suitable for putting the human in the loop to enhance robustness and trustworthiness [24, 34, 35, 44, 45].

Further related work has introduced, for example, neuro-symbolic approaches that generate textual output in the form of domain-specific languages [46, 47] or logic programs [48, 49], where the description of simple concepts detected in the input examples is the foremost goal [50–52]. Lifted Neural Networks [53] encode deep relational learning to provide insights into complex relations that go beyond the current GNN capabilities. Furthermore, there exist approaches to incorporate logic-based symbolic domain knowledge into GNNs, with the goal to improve their performance [42].

In contrast to existing work, as a first attempt, we provide a framework that applies ILP to GNN explainer output and human-expected explanations in order to validate whether the respective GNN assigned relevance to important relational sub-concepts. By applying ILP to the explainer output, we step toward providing verbal explanations for relevant graph sub-structures in the form of logic rules, thereby helping humans, such as developers and domain experts, validate the GNNs decisions against human knowledge. In particular, we want to transparently and comprehensibly evaluate the GNN’s ability to use sub-concepts that humans also find relevant. In the first step, we test to what extent ILP can learn models that faithfully find sub-concepts according to the distribution of relevance in the human-expected output of GNN explainers.

With the help of a simulated benchmark data set, we represent a human domain expert’s expectations of a GNN explainer output and provide a pipeline that transforms this output into symbolic Prolog syntax (an introduction to the syntax can be found in [8]). We then apply ILP as an interpretable machine learning approach to the symbolic input. In ILP systems, learning is usually performed by contrasting examples that belong to a target class with examples that do not belong to the target class (concept learning). In our approach, the task of the ILP component is to find commonalities in more relevant sub-graphs of a graph, while discriminating them from less relevant sub-graphs. The goal is to derive comprehensible rules that characterize the sub-concepts that may be present in an explainer’s output, that is, a set of reasons for a GNN’s decision (see Fig. 1).

Our approach is based on the assumption that GNN explainers should assign high accumulated relevance scores to sub-concepts that are known to be important for the classification task. Based on this assumption, we hypothesize that important sub-concepts will be included in ILP-generated explanations for a well-performing GNN, given that the relevance of sub-concepts learned by a GNN is taken into account in the ILP learning process. In our proposed method, the examples are graphs for which we simulate the relevance computation of a GNN explainer and which are translated into symbolic Prolog representation. The examples are divided into two sets (more and less relevant sub-graphs) based on an adaptable relevance threshold. Then, ILP learns a set of rules optimized to cover the more relevant sub-graphs and none of the less relevant sub-graphs. The rules may then serve as language-like explanations for a GNNs decision. This way, we combine relevance scores from explainers with symbolic representations in order to facilitate conceptual validation of GNNs.

The paper is organized as follows. First, we introduce our concept learning data set in Sect. 2, in particular the use case of symmetry in *Kandinsky Patterns* [54] used to benchmark our ILP-based proof-of-concept implementation. We state which outcomes the benchmark should produce accordingly. Second, we introduce the foundations of GNNs and state-of-the-art explainers for GNNs in Sect. 3; some of them inspired the design of our benchmark data set. In Sect. 4, we describe our proposed ILP-based approach that learns symbolic representations from the sub-concepts present in the most and least relevant human-expected explainer outputs. Our method is introduced as a first attempt to give insight into a GNN’s decision w.r.t. learned sub-concepts. Then, we present the experimental setting of our evaluation and the results accordingly (see Sect. 5). Finally, we conclude our work and point out future prospects in Sect. 6.

**Fig. 2:**
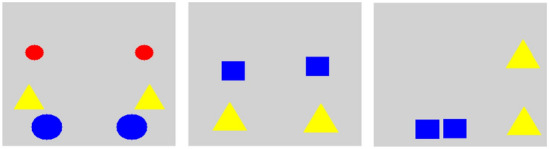
Three representative images from the vertical symmetry Kandinsky Pattern. The first one, on the left, shows an image obeying the vertical symmetry concept. The second one in the middle shows the “counterfactual” case, where the elements are slightly perturbed from vertical symmetry. The third one on the right contains a representative image from the “false” class, where the elements are non-symmetrical and are positioned far away from the vertical symmetry positions

## A Kandinsky Pattern Benchmark for Conceptual Validation

Kandinsky Pattern data sets are artificially created to test the reasoning and generalization capabilities of state-of-the-art neural networks [54]. We chose to use a Kandinsky Pattern data set as a benchmark, since the structure of some patterns is inherently relational; therefore they were first introduced in the medical domain, specifically in the classification and understanding of histopathological images [55]. There are several patterns that explore positional, arithmetic, and grouping concepts that are of inherent relational nature, mostly when the complexity increases when combined. We consider it therefore suitable for developing an evaluation framework for GNNs that may be put into practice for recognizing the characteristics in relational input, e.g., in complex medical data.

Kandinsky Patterns consist of a very simplified mathematical abstraction of histopathological images and the organizational rules that underlie them. A **Kandinsky Figure** (KF) is a square image consisting of a gray background and $n \in \mathbb {N}$ geometric objects (see Fig. 2). Each object is defined by its shape, color, size, and position within the Figure. The objects follow certain constraints; specifically, they do not overlap, are not cropped at the border of the image, and are easily recognizable and clearly distinguishable by a human observer. Given a domain of object shapes, colors, sizes, and positions, a **Kandinsky Pattern** (KP) is then the subset of all possible KFs in this domain that adhere to a certain ground truth [54]. An example of such a ground truth could be a natural language statement, such as “The figure is vertically symmetrical”. If a KF belongs to a KP, it is labeled “true”, otherwise “false”, or “counterfactual” (a slight variation of the concept that was used in the class “true”) as illustrated in Fig. 2. For our experiments, the set of contrastive figures consists of “false” cases.

Some of the more complex KPs contain areas, where elements of the same color are concentrated together. Others take into account relationships that involve objects that might be very far from each other and their spatial relations, such as “left of” and “above”.

KPs can be analyzed as images, e.g., with the help of object segmentation applied to individual KFs. Alternatively, graph extraction methods can be applied, for example, to histopathological images [56–58]; KFs can be represented as graphs, where objects represent nodes and edges express the topology of the graph accordingly.

Subsequently, such graphs can be automatically classified, e.g., by training and applying a GNN model. Then, the model’s decisions to assign a certain KP to a KF example can be explained with the help of methods that reveal which structures in a graph input have been relevant to the decision.

One KP that is considered both simple and important is **symmetry**. In this paper, we focus on this particular KP for several reasons. First, its underlying concepts can be easily defined by humans in terms of spatial, geometrical properties (e.g., “similar objects have equal coordinates along an axis that is ordinal to a mirror axis”) and can thus be easily validated. Moreover, symmetry exemplifies that relevance may have different meanings. Consider two figures, each containing similar objects: one symmetrical and one non-symmetrical constellation. In the symmetrical case, two objects would be connected by a horizontal line, whereas the line between the same objects in the non-symmetrical case would be diagonal to the mirror axis. Still, in both cases, the lines connecting the two objects may get a considerably high relevance in the classification task. The reason may be that the connecting line holds important information in both cases: horizontal or diagonal alignment. Nevertheless, in the symmetrical case, the relevance assigned to the horizontal line may be higher compared to the relevance of the line in the non-symmetrical case. There exist constellations in the KP data set, where objects in non-symmetrical figures may be connected by a horizontal line as well. It is the GNN’s task to differentiate these cases, which should be reflected in our concept-based explanations.

Note that we considered symmetry as well for the reason that it is one of the most important criteria for the diagnosis of non-cancerous vs. cancerous structures and that it is a pattern that allows for expressing gradual deformation from symmetric to non-symmetric constellations, which corresponds to the structural process a disease is causing. The detection and use of domain-specific symmetries in data and models is also a recent line of research, where GNNs play a decisive role [59].

For our benchmark, we take symmetrical and non-symmetrical KFs similar to the ones presented in Fig. 2 and transform them into a graph, where objects are represented by nodes, which in turn are connected by edges. We simulate the relevance output of a GNN explainer and assign the respective scores to the nodes’ attributes and to edges.

**Fig. 3:**
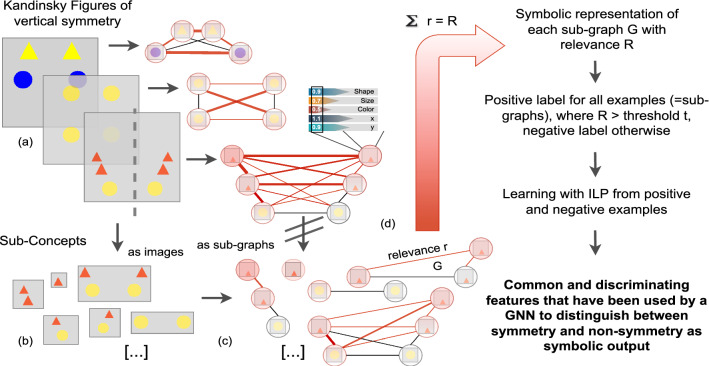
The goal of our approach: Learning symbolic representations of disjoint and common features within sub-concepts of symmetrical and non-symmetrical KFs that help to find relevant explanations for how a GNN discriminates between symmetry and non-symmetry

We thereby assume the following relationships between relevance scores and the input for well-performing GNNs. (1.) In the case of the classification decision being symmetric for a truly symmetrical figure, horizontal edges or vertical edges, and equal coordinate values of any two nodes on the symmetry axis show a higher relevance than in non-symmetrical figures. (2.) In the case of the classification decision being non-symmetric for a truly non-symmetrical figure, diagonal edges and unequal coordinates for any two nodes on an axis show a higher relevance compared to symmetrical figures. (3.) Some features of the input may get a higher relevance for both classification outcomes if they are decisive (e.g., edges connecting pairs of very similar objects compared to edges that connect very different objects). (4.) Some features, like color or shape, should be irrelevant unless the input data set itself is already not representative and a GNN would thus include irrelevant features in its decision making process.

Since explanations need to be expressive enough to induce justified trust in humans, producing verbal or language-like representations of the relations between the relevant components of a KF is desirable. Language-like expressions can be generated with the help of ILP, in the form of logic rules. Our benchmark dataset is designed in such a way that ILP should generate rules that adhere to our assumptions. To test this, we generate all possible sub-graphs for an input graph of a KF and assign relevance to the nodes and edges according to our assumptions. In this way, we realize the mapping of relevance to sub-concepts (e.g., “a horizontal connection between two objects” gets a higher relevance in symmetrical figures compared to non-symmetrical ones). The assignment of relevance was inspired by existing GNN explainers. To provide the reader with a better background, we introduce GNNs as well as GNN explainers in the next section.

## Graph Neural Networks

We shortly introduce GNNs and existing GNN explainers. Further, we describe how we intend to bridge the gap between relevance-based explainers and conceptual validation of GNNs using ILP.

### GNN Architectures

GNNs were invented to make predictions and accomplish tasks for graph-structured data. In contrast to CNNs that process grid-structured inputs in a sequential manner, GNNs operate on graph structures, not necessarily by a pre-specified order. The tasks that they can effectively solve are node classification, link prediction, and graph classification.

One of the first architectures, the Graph Convolutional Network (GCN) [60], took inspiration from CNNs to accomplish tasks, meaning that information about each element is computed by aggregating and combining information from neighboring elements. By the same means as the grid-structured neighborhood of a pixel is used to compute the filter values of a CNN, the k-hop neighborhood of a node is utilized and updated during the training process.

Equation 1 describes the main operations performed in a GNN that update the values of the initial features *h* on nodes and edges in a manner that is guided by training. *k* is the number of aggregation iterations and corresponds to the number of hops in the neighborhood $\mathcal {N}$ that will be considered. The aggregation operation (Eq. 1) uses all features of the neighboring nodes (denoted with *u*); those can be of any form and can numerically encode several characteristics like size, color, shape and many more.

1 $$\begin{aligned} \begin{aligned} a_v^{(k)}= & {} \text {AGGREGATE}^{(k)} \Big ( \Big \{ h_u^{(k-1)}: u \in \mathcal {N}(v) \Big \} \Big ) \\ h_v^{(k)}= & {} \text {COMBINE}^{(k)} \Big ( h_u^{(k-1)}, a_v^{(k)} \Big ) \end{aligned} \end{aligned}$$

After the aggregation operation, the combine operation provides the new values for the features of node *v* (equation 1), which are typically called embeddings.

Those two operations are used by GNNs to solve all possible tasks; depending on the task, researchers have found that GNNs can be extended by functionality, having different purposes and serving different goals [60]. Particularly the Graph Isomorphism Network (GIN) architecture learns a function with the use of Multi-layer Perceptrons (MLP) [3]. This provides the necessary flexibility for injectiveness, for the maximum possible discrimination ability, as well as the property of mapping similar graph structures to nearby embeddings. Graph classification with the use of this architecture could succeed in separating different KP classes. xAI methods uncovering the GNN’s decision-making process could help researchers see if those decisions are based on elements that make humans understand the causal factors of a decision [61].

### Explainability of Graph Neural Networks

Several methods have been proposed to uncover the underlying decisions made by a GNN classifier by computing relevance scores for nodes, edges, features thereof, walks and sub-graphs. They provide the means toward transparency and, ideally, could be used in combination with symbolic approaches to generate verbal or language-like explanations for increased comprehensibility and better control of the overall system to satisfy the validation and improvement requirements of human-centric AI [10, 35, 45].

However, currently, none of the existing GNN explainers generates verbal or language-like explanations and the vast majority of them search for the substructure that has the highest relevance according to specially designed losses, metrics, and assumptions. In the following paragraphs, we give a short overview.

**GNNExplainer**: For node as well as graph classification tasks, the GNNExplainer is used to identify important sub-graphs in the input graphs that are going to be explained [17]. Graph and node features are masked; the search for an adequate mask is performed by an optimization algorithm. If the substructure has a high mutual information value [62] w.r.t. the predicted label distribution, then it is a good enough explanation. A variant of this method, the **PGExplainer** [63] uses a deep neural network to parameterize the generation process of the masks and the explanations in general, thereby explaining multiple instances of sub-graphs at the same time.

**GNN-LRP** [16]: is a method that applies the Layer-wise Relevance Propagation (LRP) principle [64] to GNN architectures that fulfil the required constraints. The main strategy of LRP, namely the backpropagation of relevance from the output to the input, layer-by-layer, reflecting the intensity of the learned weights in proportion to each other, is transferred to the GNN message-passing scheme (see section 3.1). By applying Taylor decomposition w.r.t. to the graph’s Laplacian matrix, the relevance of each possible walk of a particular size in the graph is computed. This method, although time and memory-consuming, has several benefits that go beyond the fact that it computes both positive and negative relevance. By returning a relevance for each walk, the “flow” of the relative importance of a node is captured in the relevance of its neighboring nodes.

**MotifExplainer**: [65] is trying to find motifs (sub-graphs that may be characteristic, e.g., circles) in the computation graph of each node that is classified. The domain knowledge is incorporated to reduce the search space effectively by selecting motifs that are known to play a role in the prediction; this procudes explanations that have both high fidelity and sparsity. Those motifs are fed into the model, their embeddings, as well as the performance of the model, is registered and an additional attention model provides the weights by which sub-graphs are selected. The motifs that have a similar prediction performance and have a high attention weight are the ones that are characterized as relevant [14].

**GraphLIME**: [18], in the same means as LIME [66], tries to find a local surrogate model to explain each node. The N-hop neighborhood of a node is sampled to compute the most representative features, which then serve as the explanations of the prediction.

**PGMExplainer**: The PGM-Explainer is one of the first counterfactual xAI methods for GNNs [19]; the main goal is to answer the question “what if” the input was different. Counterfactuals are usually created by adding or removing features from the input and this creates a broad spectrum of possibilities on graph data sets. Perturbing the topology of a graph in a way guided by the change in performance leads to the computation of a Probabilistic Graphical Model (PGM) [67, 68] with the help of structure and parameter learning. The resulting PGM’s random variables (also expressed through their conditional probability tables) determine the distribution of values that node features can take.

**XGNN**: This method uses Reinforcement Learning to guide the search toward the substructure in the graph that was relevant to the decision [69]. It takes into account prior knowledge by starting with an initial state - a small sub-graph - and trying to find the substructure that will keep the prediction performance intact by exploration and exploitation, but also selects graphs that are in accordance with domain knowledge.

### Toward Verbal and Trustworthy Explanations

While metrics exist to measure the efficiency of an explanation in an algorithmic way, such as fidelity, we believe that for humans, verbal explanations may provide insight into a GNNs decision-making in the most natural way. Furthermore, verbal explanations could be utilized to validate the graph explainer itself. The graphs extracted from symmetrical and non-symmetrical KFs provide an ideal benchmark for such experiments, as they can be categorized into sub-concepts (the sub-graphs with attributes) and verbally described by a human.

In our benchmark, we put a special focus on simulating the expectations of a human user w.r.t. explanations for GNNs. For this particular data set, the input graph is perturbed in all possible ways and while assuming a correct GNN prediction, the human prospects for the relevance values for nodes, edges, and sub-graphs are computed. We chose this approach strategically to enable human-in-the-loop valuable input in a post-hoc procedure, rather than requiring the human to mask the extracted input graph according to domain-specific concepts. Allowing the explainer to only perturb features with or without a concept would not be feasible since it would require modifying each explainer at its very core. This would not be an adequate step toward a general tool for the validation of any explanation inferred by any relevance-based explainer.

In this work, we explore and investigate a new strategy to map human-masked concepts to a learnable model parameter (here: symbolic predicates) inferred by an ILP system. This is done by decomposing the input graph into sub-graphs that contain or do not contain the sub-concepts of interest. After each decomposition, relevance scores are re-calculated for our simulated explainer. We argue that the distribution of relevance scores should remain robust to these concept-wise perturbations and thus will converge to a model parameter.

As a first investigation, we use a synthetic data set with hypothetical relevance scores to validate the general feasibility of our approach. Once the general applicability of the presented approach is fully validated, we will explore relevance scores that are produced by the explainers described in Sect. 3.2.

## Combining Relevance Scores with Symbolic Predicates

As motivated in the introduction of this paper, ILP is an interpretable machine learning approach suitable for generating language-like, expressive explanations. It has been applied to explainer output in previous works to introduce relational learning for explaining predictions of CNNs [35, 37]. In this work, we integrate ILP to create a comprehensible validation framework for graph classification with GNNs.

In this section, we first give an overview of our approach. We then explain how our benchmark data set is created and which assumptions underlie its design. Subsequently, we introduce ILP as a concept learning approach and how it can be applied to derive comprehensible, language-like rules composed of symbolic predicates that may characterize the sub-concepts that are present in an explainer’s output, that is, a set of reasons for a GNN’s decision. Finally, we state our hypotheses w.r.t. results we expect from applying our approach.

### From Relevance to Sub-Concepts

The usual GNN-based graph classification pipeline, including the explanation of predictive outcomes, would basically consist of three steps. First, the GNN is trained and tested on pre-labeled graph-structured input examples to achieve the highest possible classification performance. Second, the learned GNN model is ready to be applied to yet unseen graph-structured examples from the same domain in order to derive class labels. In the third step, developers and decision-makers can ask for explanations for individual classified examples. These explanations are computed by GNN explainers and their extensions (e.g., ILP as an extension to produce comprehensible output for GNN explainers). Recall Fig. 1 for an overview.

Our work has two branches as far as relevance distributions are concerned. First, we use human domain knowledge for modeling the expected relevance values, and second, we’ve already started analyzing GNN explainers, as presented in Sect. 3.2. The relevance values comprise the basis for the symbolic representation and input to ILP after transformations that will be clarified in Sect. 5. As presented in Fig. 1, our ultimate goal is to compare the explanations expected by humans and the ones created by explainers and thereby achieve concept validation.

Figure 3 illustrates our benchmark, that is based on KFs of vertical symmetry. KFs are depicted on the left side, and the respective explainer output for their graph representation is on the right side of part (a). Each object (yellow triangle, blue circle, etc.) is represented as a node, which is connected through edges to all the other nodes. The red coloring of nodes and edges illustrates the value of relevance assigned by a GNN explainer or as given by humans. The more intense the red color is, the higher is the relevance of the component. Attributes of objects, like shape, size, color and the coordinates of the center-point of an object, are assigned as well with relevance values. In order to derive the relevance for sub-concepts and not for the whole graph of a KF, we decompose each example. Part (b) illustrates the human-understandable sub-concepts (e.g., “a single red triangle”, “two yellow circles side by side”, etc.). At this point, we want to point out that ILP is capable of generalizing from concrete attribute values by introducing variables, making it possible to express more abstract sub-concepts like “some objects side by side”. Part (c) depicts the sub-graphs derived from decomposed KFs and assigned with relevance based upon our assumptions as described in Sect. 2. Since our assumption is that important sub-concepts aggregate more relevance, we summarize the relevance *r* for each sub-graph *G* to derive its total relevance *R*. We then transform the sub-graphs with relevance *R* into a symbolic representation in Prolog syntax (see part (d)). An example can be found in Fig. 5.

Note that our goal is not to learn an interpretable surrogate model for a GNN with ILP, as presented in [37], but to identify the sub-concepts that are present in the more relevant sub-graphs in contrast to the less relevant sub-graphs. This is a different type of learning task that is not based on splitting symmetrical and non-symmetrical examples. It takes into account that structures that are present in the most relevant sub-graphs from symmetrical and non-symmetrical KFs do not have to be disjoint. This applies, for example, to the edge between two horizontally located nodes that have the same y-axis position in case of symmetry or not in case of non-symmetry. In both cases, the edge holds important information about the class: it is either a horizontal or a diagonal line.

As outlined on the right side of Fig. 3, ILP learns rules from the set of all sub-graphs (represented in Prolog) that apply only to the most relevant sub-graphs. Thus, each rule may contain a symbolic representation of sub-concepts in the form of so-called predicates to learn symmetry and non-symmetry.

To generate the input set for ILP, we rank sub-graphs according to their relevance sum. We then divide the sub-graphs w.r.t. their rank at a threshold. A justified criticism of this approach is that small sub-graphs may have a lower total relevance than bigger sub-graphs. We address this in our benchmark by keeping the total relevance more or less constant among important sub-graphs and distributing it over the structures that represent important sub-concepts. Note that for real explainer output, normalizing the relevance in advance to ranking would be necessary. Sub-graphs that have a relevance sum above a configurable threshold *t* are considered members of the class of “more relevant” examples; the rest is considered “less relevant”. By learning from such examples with ILP, we generalize over symbolized sub-graphs of symmetrical and non-symmetrical KFs. This way, we aim to find the commonalities in the most relevant sub-concepts while discriminating them from the least relevant sub-concepts, both for symmetrical and non-symmetrical examples.

### A Generated Benchmark for Vertical Symmetry

**Fig. 4:**
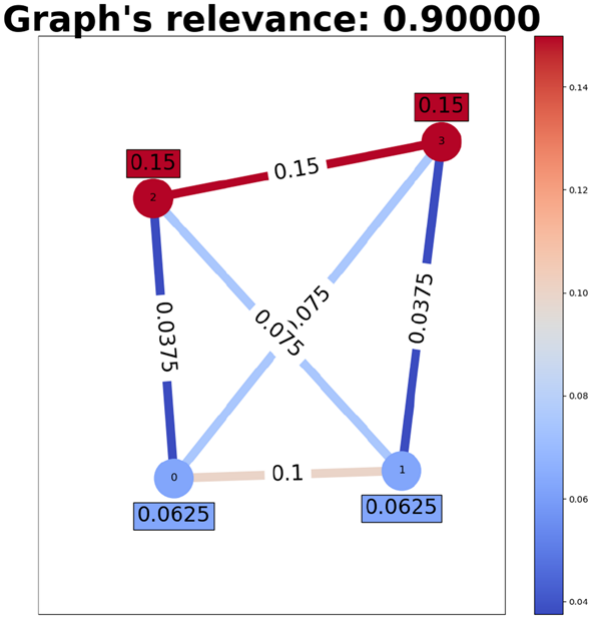
The fully connected graph for four objects with the pre-specified relevance per node and edge in a non-symmetric case. The relevance of the whole graph is the sum of the relevance of all its components. The relevance spectrum has values from 0 to 1 (from blue to red)

For the validation of GNNs and GNN explainers with the help of ILP, we provide a benchmark data set based on the KP for vertical symmetry. Every image in the data set contains either a vertically symmetric constellation of objects or not. Currently, our benchmark includes graphs with one to four objects which have a predefined size, color, and shape, e.g., one triangle and one circle left of the mirror axis and similarly on the right. We created both symmetric and slightly perturbed cases as far as the positions of the objects are concerned. In particular, to create non-symmetrical constellations, each of the coordinates of the four nodes is a noisy version of the corresponding coordinates in the symmetric case. The added noise is sampled eight times in total (twice for each node, one for each coordinate) from a normal distribution $\mathcal {N}(\mu , \sigma ^2)$ with $\mu =0$ and $\sigma = 0.1$. The benchmark generator makes sure that the produced objects do not overlap and that they have distinct relations to each other, as presented in Fig. 2. We consider all possible sub-graphs that can be generated from input graphs with one, two, three, and four objects. For each set of objects, we compute every possible graph with every possible combination of edges. We further include all possible sets of nodes that are not connected by edges.

The total relevance *R* of each sub-graph *G* can range from 0 to 1 and is the sum of all relevance values of nodes and edges $\in$ *G*. The relevance distribution inside *G* is defined with the use of domain knowledge about sub-concepts that may be important for differentiating between symmetry and non-symmetry from a human’s perspective. The following principles were applied: First, the *R* is non-uniformly distributed within a sub-graph to account for the fact that not all components may be equally important, especially in larger sub-graphs. This is preserved even for sub-graphs, where important sub-concepts occur multiple times, under the assumption that GNNs weigh features efficiently and not for the sake of completeness. Second, in accordance to our assumptions presented in Sect. 2, horizontal edges or vertical edges, as well as equal coordinate values of any two nodes on the symmetry axis get a higher *R* in symmetrical figures compared to non-symmetrical ones. Diagonal edges and unequal coordinates for any two nodes get a higher *R* in non-symmetrical figures compared to symmetrical ones. Further, some edges get a higher *R* in both cases if the connection they express is independently important, e.g., a line between a pair of objects (being horizontal in symmetry and diagonal in non-symmetry). Sub-graphs that contain no edges and where the GNN would rather consider the size, shape, or color of an object, are assigned with a lower *R* in order to account for the considerable irrelevance of such features w.r.t. symmetry. Of course, symmetry holds only if the objects on the symmetry axis are equal in appearance, but in general, concrete size, shape or color values should not play a role. The distribution of relevance in a sub-graph is exemplified in Fig. 4. It shows a non-symmetrical example in which edges that are nearly horizontal get a higher relevance than others.

We further create sub-graphs on four levels of expressiveness named *single objects*, *x-axis*, *y-axis*, and *alignment relations*. In the case of single objects, all object constellations are created without any edge connecting them - practically all graphs with no edges. In the x-axis case, all possible graphs are created. However, the connection between two nodes is only kept if it expresses a relation w.r.t. the x-axis (“left-of” and correspondingly “right-of”). Similarly, for the y-axis case, only the “down-of” and “up-of” relations are kept. For the alignment case, the eight spatial relationships are: “top-left”, “top-middle”, “top-right”, “middle-left”, “middle-right”, “bottom-left”, “bottom-middle”, and “bottom-right”. To compute them, each object in an image takes an imaginary center position, and the object connected to it, is either inside (contained in) one of the eight sectors or it will be found on one of the eight lines that separate the sectors. It is obvious that if a particular relation is found between two objects, then the corresponding reverse relation must be detected for the same two objects. The code to generate and re-use our benchmark can be found in the corresponding GitHub repository.^1^

### Learning Symbolic Predicates with ILP

ILP can be applied to symbolic input to induce a set of logic rules that separates a target class from contrastive classes. Most ILP frameworks perform concept learning, meaning induction based on a set of so-called *positive* examples, belonging to the target class, and a set of so-called *negative* examples, not belonging to the target class. The input data, the algorithm as well as the learned set of rules is represented as logic programs that are interpretable for humans [35]. To be more specific, ILP is based on first-order logic, and program code is represented in Prolog, which is a declarative programming language. We shortly summarize the key terminology of ILP and Prolog based on the introduction given in [44].

Prolog programs consist of a finite set of Horn clauses. A Horn clause is a set of disjunctive literals with at most one positive literal and can be represented as an implication, written in the form

$$\begin{aligned} A_0 \leftarrow A_{1} \wedge \ldots \wedge A_{n},\ \text {where}\ n \ge 0. \end{aligned}$$

Each literal $A_i$ has the form $p(t_1,\ldots ,t_m)$, consisting of a predicate *p* and terms $t_i$. Terms are either a constant symbol, a variable or a composite expression. The literal $A_0$ is called the *head* and the conjunction of elements $\bigwedge _{i=1}^n A_i$ is called the *body* of a clause. In Prolog, implication $\leftarrow$ is denoted by $:-$ and the colon represents conjunction $\wedge$. Clauses end in a full stop.

Basic Prolog concepts include facts, rules and queries. Horn clauses with an empty body are denoted as *facts* and express unconditional statements, otherwise they are called *rules* and express conditional statements. Semantically, we can read a clause as “the conclusion (or *head*) $A_0$ is true if every $A_i$ in the body is true”. Facts are literals with constant terms. Rules express a logical implication to describe that a condition holds if a combination of literals holds, supported by given facts. *Queries* can be used to retrieve information from a Prolog program. Queries can be either facts, to check for their truth or falsity, or can be composite expressions to retrieve terms that make those expressions true. Since we do not apply queries directly in our approach, we focus on facts and rules in the following paragraphs.

To learn symbolic rules that contain relevant sub-concepts to explain the classification of KFs, we applied the ILP framework *Aleph* [70]. Aleph uses inverse entailment to induce a theory, consisting of Prolog rules, from a set of positive and negative examples and background knowledge that describes these examples. The default algorithm performs four steps. First, it selects an example to be generalized. Second, it constructs a most specific clause based on a pre-defined syntactical restriction. Then, a clause that is more general than the most specific clause and which entails the selected example is found, and finally, all the examples that are covered by the more general clause (rule) are removed from the set of candidate examples to be generalized.

**Fig. 5:**
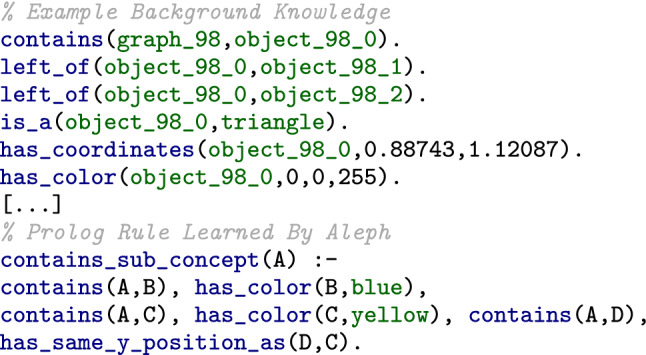
Prolog representation of an exemplary KF sub-graph (top) and of a Prolog rule that applies to it (bottom)

Figure 5 serves as an illustrative example for our KP benchmark. At the top, it contains an excerpt from the so-called background knowledge, describing one sub-graph of a KF represented by symbolic facts. The facts express which objects are contained in a graph (both denoted by their respective identifiers) and which spatial relationships hold between the contained objects (e.g., “left_of”) as well as the attributes in terms of coordinates (denoted by “has_coordinates”), shape (d. b. “is_a”) and color (d. b. “has_color”) of the objects. We omit the relevance values for all predicates, except for the relevance of the whole graph (d. b. “has_relevance”). These relevance values are excluded from the ILP learning process since the relevance is solely used to split the examples into positive and negative ones. At the bottom, Fig. 5 shows a rule that may be learned by Aleph to discriminate between more relevant and less relevant sub-graphs. It is a rule that covers only vertically symmetrical examples. It represents the sub-concept of equal y-position of contained objects. It may also contain rather irrelevant sub-concepts like the color of an object.

### Expected Explanations for Symmetric Kandinsky Pattern Classification

In accordance with our assumptions presented in Sect. 2 and the design of our benchmark that is in line with these presuppositions (see Sect. 4), we expect that ILP learns a set of rules, some of which may cover only symmetrical examples or non-symmetrical examples and where some may cover both. For vertical symmetry, we expect ILP to consider equal y coordinates in objects as well as horizontal and vertical connections between such objects, while for the non-symmetrical examples we expect ILP to consider diagonals between objects with different y coordinates. We further expect that there might be some sub-concepts, like the shape and color of objects that might be included for symmetry if they are equal for different objects and which might be included for non-symmetry if they are not equal.

## Evaluation

In this Section we first describe the experimental setting we used and the obtained results.

### Experimental Setting

For our experiments, we distinguish four levels of expressiveness, as presented in Sect. 4. We want to test different levels of detail in the background knowledge for representing sub-graphs of the original graph and, thus, sub-concepts.

**Fig. 6:**
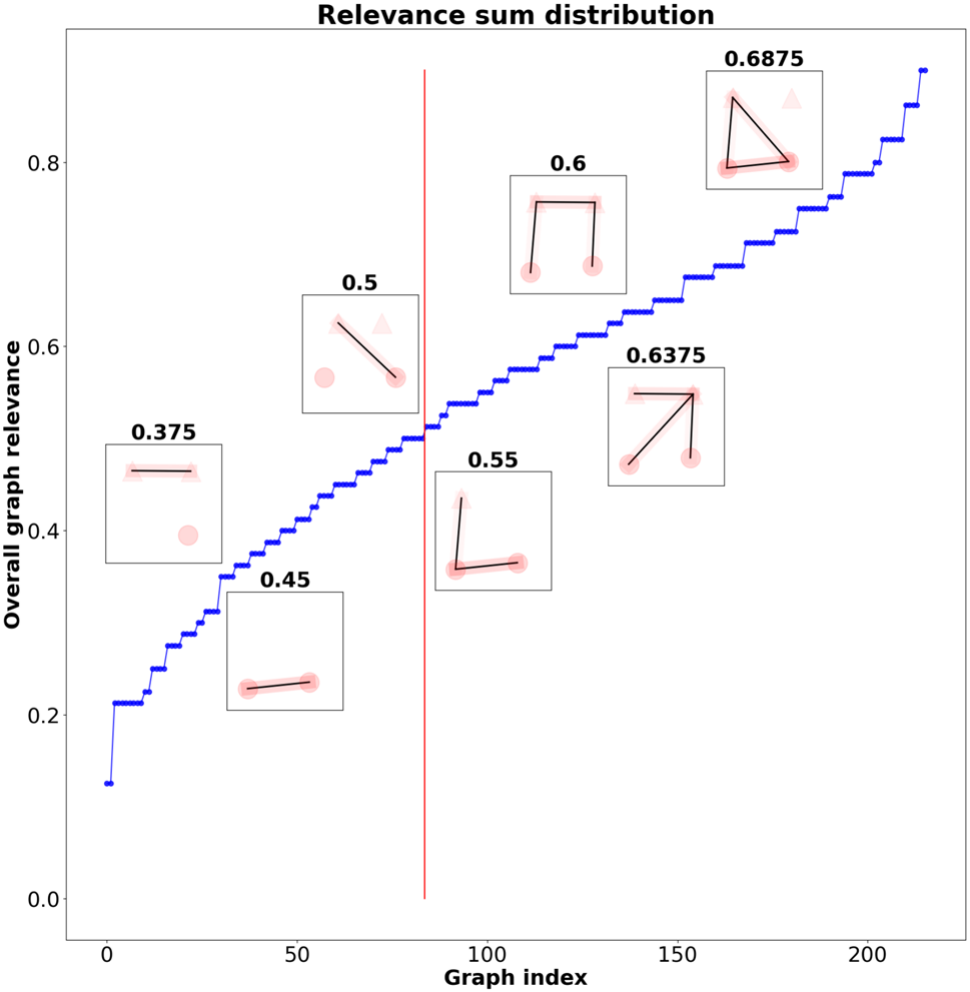
Plot of the sorted human-expected relevance, as well as some characteristic sub-graphs. The red vertical line represents the threshold *t* that divides the graph data set into more and less relevant sub-graphs (here, at *t* = 0.5)

**Fig. 7:**
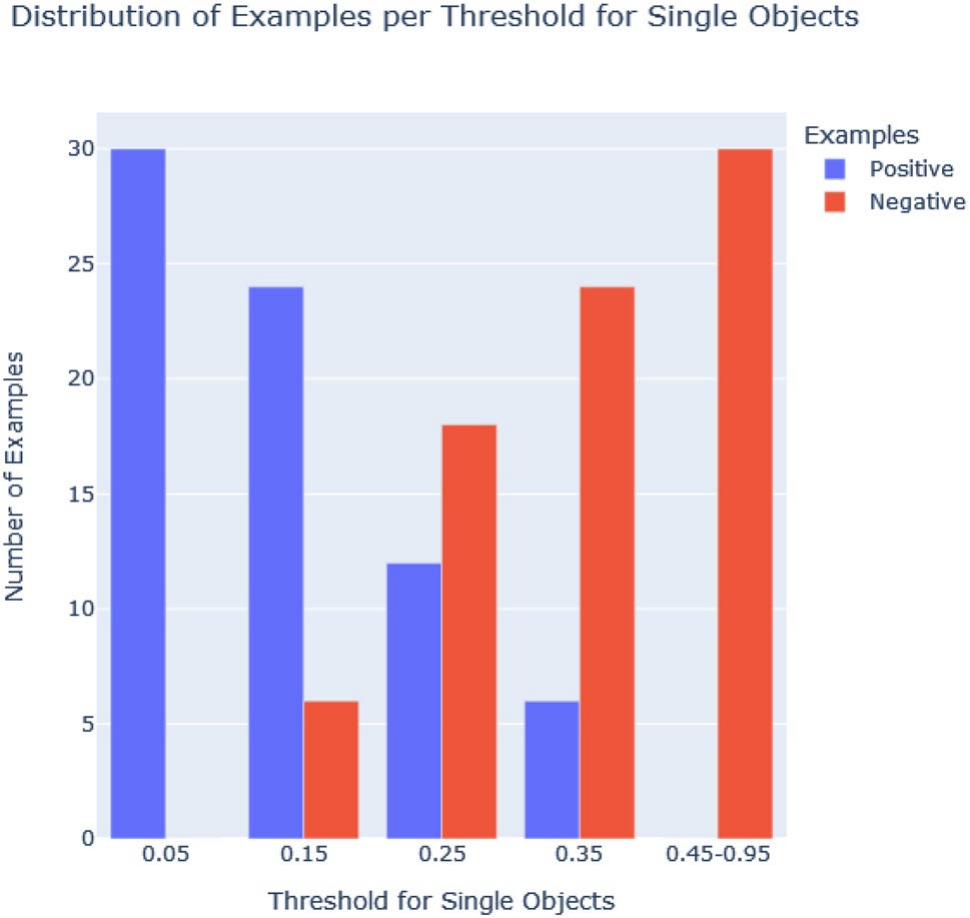
The number of positive and negative examples per threshold for single objects

**Fig. 8:**
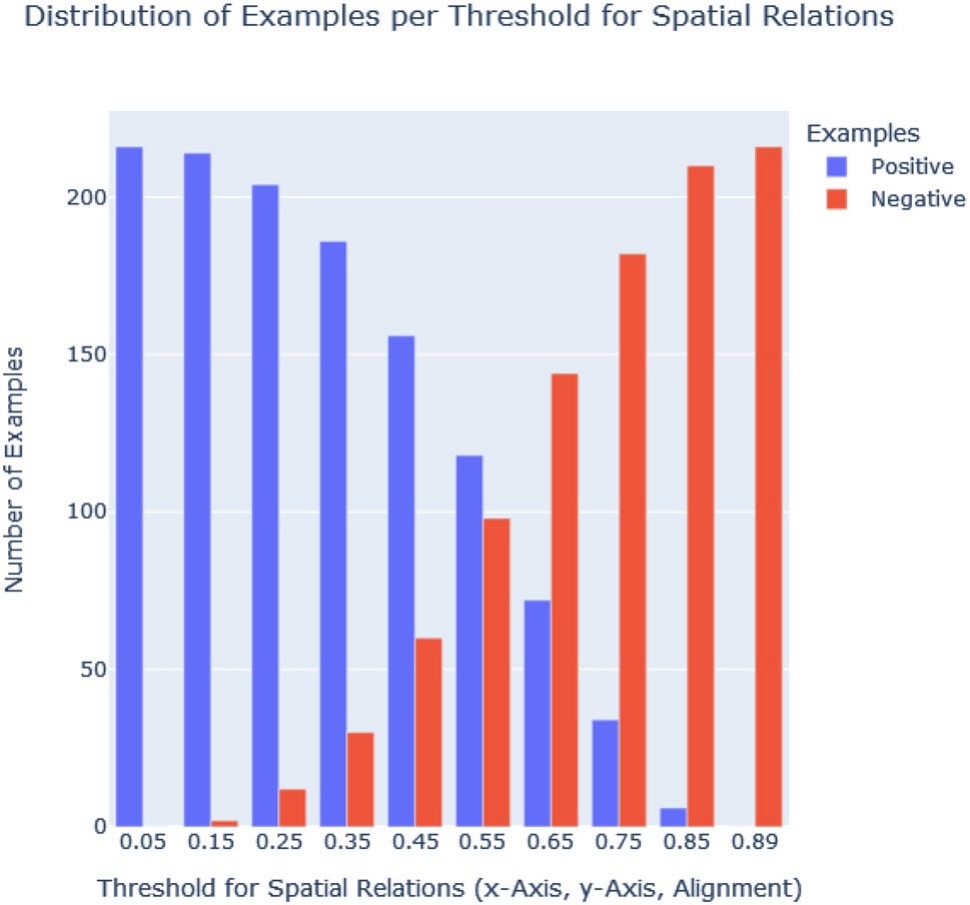
The number of negative and positive examples per threshold for spatial relations

Determined by the number of possible combinations, the set of input examples is 20 for single objects and 216 for the x-axis relational case, the y-axis relational case and the more advanced spatial alignments. We set the relevance threshold to a particular value and split the input examples in order to assign them either to the target class or the contrastive case: the ones with less relevance than the threshold value (negative examples) and the ones with equal or more relevance (the positive examples) than the threshold. Figure 6 shows an exemplary split at a threshold of 0.5. We performed experiments for different relevance thresholds - ranging from a minimum threshold of 0.05 (meaning 5 % of a maximum relevance of 1) to the maximum threshold of 0.95 - to test Aleph’s robustness against imbalanced input data and its generalization capabilities. Different thresholds lead to a different number of positive and negative examples and, thus, to imbalanced input. Figure 7 shows the distribution of examples according to the threshold for single objects, whereas Fig. 8 depicts the distribution for spatial relations (along the x-axis, y-axis, and as alignments). As expected, the threshold regulates the balance or imbalance of the input data set for Aleph. This is in line with the fact that relevance values computed by GNN explainers (see 3.2) are generally not equally distributed over sub-graphs. This valuable property is used to explore the capabilities of Aleph to learn from small and potentially imbalanced data sets.

The unequal distribution of relevance values puts the generality of explanations produced by ILP under test. Therefore, we examine the robustness of explanations dependent on the distribution of the relevance across sub-graphs and the chosen threshold. We observe that the best balance for single objects is at *t* = 0.25 (see Fig. 8), for relations, it is 0.55 (depicted in Fig. 8). In the case of single objects, a split of examples at a threshold of 0.05 yields no negative examples, whereas a threshold of 0.35 yields no positive examples. Similarly, for relations, the corresponding thresholds are 0.05 and 0.85. This is in accordance with the fact that graphs do not accumulate enough relevance at higher thresholds.

### Results

**Fig. 9:**
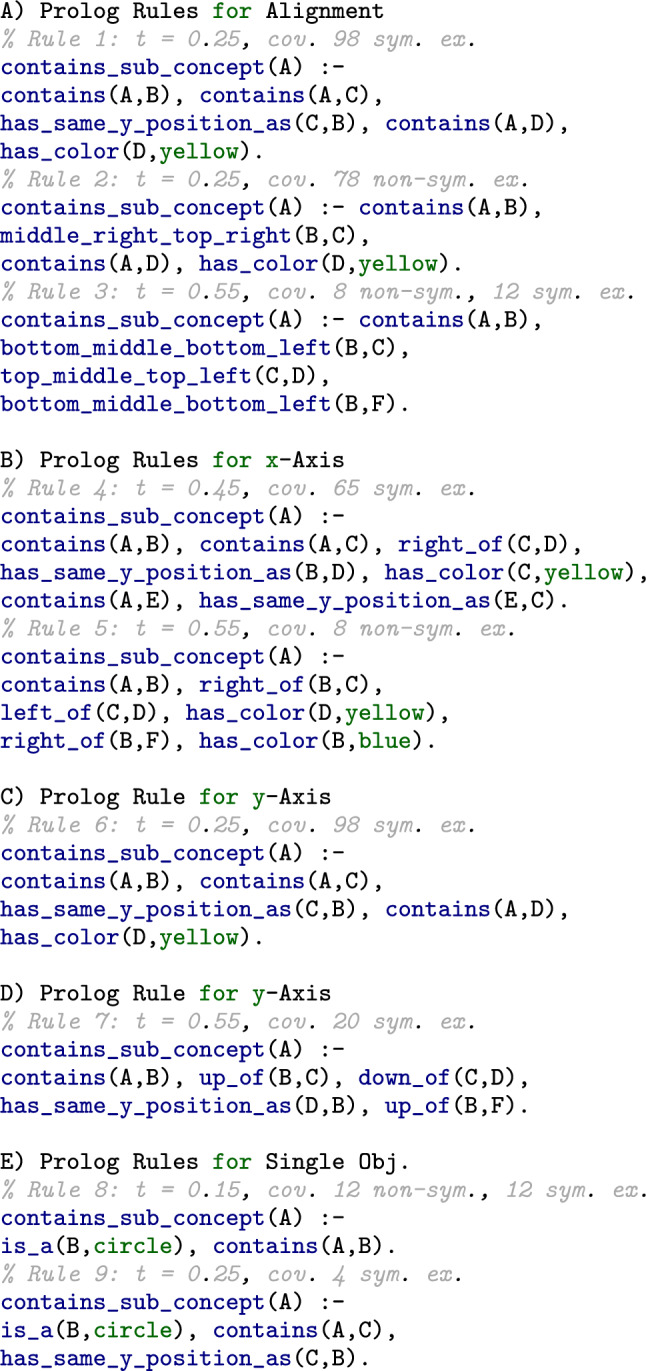
Exemplary rules learned for each use case (Single Objects, x-Axis, y-Axis and Alignments)

This section presents the results for each of our four levels of expressiveness. In particular, we list some rules that had the highest number of covered positive examples while covering as few negative examples as possible and some interesting cases that demonstrate the suitability of learned symbolic predicates for validation.

Figure 9 lists nine exemplary rules that stood out from our results. The first rule was learned for spatial alignment at the threshold of 0.25. Although there was just a small number of contrasting examples (see Fig. 8), the rule is quite representative for symmetrical examples (covering 98 of them) by containing a predicate that imposes the condition of the same y-coordinate value on two related objects, C and B. The rule further states that the graph must contain an object D that is yellow. Something similar applies to the second rule that is learned for the same use case for sub-graphs from non-symmetrical examples. There is a yellow object E; in contrast to the first rule, the predicate expressing the spatial relationship describes a diagonal (B is middle-right top-right of C). Another interesting rule is the third one. Here, ILP found a set of predicates that covers symmetrical as well as non-symmetrical examples, where the sub-graphs had a total relevance higher than the threshold of 0.55. The rule contains several diagonals, e.g., the diagonals top-middle-top-left and bottom-middle-bottom-left. Comparing rules 4 and 5 that were learned for x-Axis relations, one for a threshold of 0.45 and the other for a threshold of 0.55, we can see that rule 4 which covers only symmetrical examples, contains a predicate for the same y-coordinate again. Rule 5, on the other hand, covers only non-symmetrical examples and contains no predicates that are representative of symmetry. Instead, the colors of objects seem to provide a more general pattern here by being different (yellow, blue). Rules 6 and 7 were both learned for y-Axis relations. Their comparison shows how important it is to balance positive and negative examples, meaning examples with higher relevance and those with lower relevance, in order to avoid noise. For rule 6, which was learned at a threshold of 0.25, a total of 98 symmetrical examples could be covered. However, the rule contains a rather irrelevant restriction to the color yellow for an object. In contrast to this, in rule 7, which was learned at a threshold of 0.55 (which is the most balanced case for relations; see Fig. 8), no statements about color are included. Instead, only predicates of containment and spatial relations were induced. For the single objects, we selected two interesting rules. While rule 8 was learned for 12 symmetrical as well as 12 non-symmetrical examples, rule 9 covered only four symmetrical examples. The first one includes information, such as the kind of shape (here: circle), and the second one contains a statement about the same y-coordinate again.

Our results show that aligning the relevance threshold with the distribution of relevance in the sub-graphs produces rules that contain symbolic predicates that match with human-expected sub-concepts.

## Discussion and Conclusion

The results in the previous section show that symbolic predicates can be learned that describe the sub-concepts that are representative of for a set of sub-graphs, either for symmetrical or non-symmetrical examples or both. We have learned that symmetrical and non-symmetrical examples are often covered by separate rules, even though they share the positive label, which is a precondition to find sub-concepts that distinguish both groups. We could observe that the investigation of positive and negative labels provides information about the relevance distribution of the explanations. The rules, on the other hand, may indicate whether there is noise due to over-represented, rather irrelevant sub-concepts. At the same time, symbolic rules that cover a high number of examples in a rather balanced setting of positive and negative examples may be representative of the concepts that are underlying the decision of a GNN, given that the explanations are gathered from a GNN explanation method. Currently, we have applied our approach solely to human-expected relevance scores. In the future, we will take into account the model accuracy of a GNN (where low accuracy may be a driver for noise in the sub-concepts) as well as the output of real explainers (which may cause imbalanced input data). We’ve already started experiments using a Graph Convolutional Network (GCN) [60] and currently, investigating the output of different state-of-the-art explainers. In order to mitigate noise and to improve a GNN model by a human-in-the-loop, a corrective feedback technique could be integrated similar to previous work [35].

Our benchmark data set could be utilized in additional experiments to compare the correctness, similarities, and differences in explanations by humans and AI systems, as well as for an investigation on how a human could learn from comprehensible AI. Our presented approach is a first attempt toward generating comprehensible output for relevance-based methods (as presented in Sect. 3.2) to explain a GNN’s classification. For this, a rigorous comparison of different explanation methods as well as data sets is necessary. While we applied our approach to the Kandinsky Pattern of vertical symmetry, future work could include further *relational* data sets and examples. A comparison of GNNs with ILP models by means of classification performance and interpretability could be another interesting investigation. We further note that our approach is a combination of model-specific and model-independent methods w.r.t. GNN architectures. While relevance-based explanations are computed based on the inner workings of a specific GNN architecture, ILP allows for conceptual abstraction. That is, our approach can be applied to any explainer that computes a linear or ordinal score by means of relevance for features that can be easily modified, such as nodes, edges, and attributes. Our implementation is the very first step toward comparing different GNN explanations and, consequently, their symbolic representation. In the future, research will also include user studies in order to verify our approach. Ultimately, we intend to further extend our proposed technique toward actionable, interactive systems by combining GNN, xAI, and a logical approach that is understandable to humans.

## Footnotes

1 GitHub repository HuCE (Human-centered explanations): https://github.com/asaranti/HuCE---Human-centered-Explanations.

## References

[1] Ravid Shwartz-Ziv and Naftali Tishby. Opening the Black Box of Deep Neural Networks via Information. Information Flow in Deep Neural Networks, page24, 2022

[2] Zhou J, Cui G, Shengding H, Zhang Z, Yang C, Liu Z, Wang L, Li C, Sun M (2020). Graph neural networks: a review of methods and applications. AI Open.

[3] Keyulu X, Weihua H, Jure L, Stefanie J (2018) How powerful are graph neural networks? In: International conference on learning representations

[4] Gabriel V (2002) Algorithms on Trees and Graphs, 112. Springer, New York

[5] Horst B, Bruno TM (1993) Similarity measures for structured representations. In: European workshop on case-based reasoning, pp 106–118, Springer

[6] Zonghan W, Pan S, Chen F, Long G, Chengqi Z, SYu P (2020). A comprehensive survey on graph neural networks. IEEE Trans Neural Netw Learn Syst.

[7] Kriege NM, Johansson FD, Morris C (2020). A Survey on Graph Kernels. Applied Network Science.

[8] Luc De R (2008) Logical and relational learning. Springer, New York

[9] Xiao-Meng Z, LiLiang LL, Ming-Jing T (2021) Graph neural networks and their current applications in bioinformatics. Front Genet 12 10.3389/fgene.2021.690049 PMC8360394 34394185

[10] Holzinger A, Malle B, Saranti A, Pfeifer B (2021). Towards multi-modal causability with graph neural networks enabling information fusion for explainable ai. Inf Fusion.

[11] Yu Z, Haixia Z, Xin H, Shufeng H, Dengao L, Jumin Z (2022) Graph Neural Netw Taxonomy Adv Trends 13(1)

[12] Andreas Holzinger, Anna Saranti, Christoph Molnar, Prezemyslaw Biececk, Wojciech Samek (2022) Explainable ai Methods - A Brief Overview. In XXAI - Lecture Notes in Artificial Intelligence LNAI 13200, pages 13–38. Springer,

[13] Gesina Schwalbe, Bettina Finzel (2021) Xai Method Properties: A (Meta-) Study. arXiv preprint arXiv:2105.07190,

[14] Hao Yuan, Haiyang Yu, Shurui Gui, Shuiwang Ji (2020) Explainability in Graph Neural Networks: A Taxonomic Survey. arXiv preprint arXiv:2012.15445 10.1109/TPAMI.2022.3204236 36063508

[15] Hao Yuan, Jiliang Tang, Xia Hu, Shuiwang Ji (2020) Xgnn: Towards Model-Level Explanations of Graph Neural Networks. In Proceedings of the 26th ACM SIGKDD International Conference on Knowledge Discovery & Data Mining, pages 430–438,

[16] Thomas Schnake, Oliver Eberle, Jonas Lederer, Shinichi Nakajima, KristofT Schütt, Klaus-Robert Müller, and Grégoire Montavon (2020) Higher-Order Explanations of Graph Neural Networks via Relevant Walks. arXiv preprint arXiv:2006.03589 10.1109/TPAMI.2021.3115452 34559639

[17] Ying R, Bourgeois D, You J, Zitnik M, Leskovec J (2019). Gnnexplainer: Generating Explanations for Graph Neural Networks. Adv Neural Inf Process Syst.

[18] Qiang Huang, Makoto Yamada, Yuan Tian, Dinesh Singh, Dawei Yin, YiChang (2020) GraphLIME: Local Interpretable Model Explanations for Graph Neural Networks. arXiv preprint arXiv:2001.06216v1,

[19] MinhN Vu, MyT Thai (2020) Pgm-Explainer: Probabilistic Graphical Model Explanations for Graph Neural Networks. arXiv preprint arXiv:2010.05788

[20] PhillipE Pope, Soheil Kolouri, Mohammad Rostami, CharlesE Martin, Heiko Hoffmann (2019) Explainability Methods for Graph Convolutional Neural Networks. In 2019 IEEE/CVF Conference on Computer Vision and Pattern Recognition (CVPR), pages 10764–10773,

[21] Lapuschkin S, Wäldchen S, Binder A, Montavon G, Samek W, Müller K-R (2019). Unmasking Clever Hans Predictors and Assessing What Machines Really Learn. Nat Commun.

[22] Bastian Pfeifer, Anna Saranti, Andreas Holzinger (2021) Network Module Detection From Multi-Modal Node Features With a Greedy Decision Forest for Actionable Explainable ai. arXiv preprint arXiv:2108.11674,

[23] Marc Hanussek, Falko Kötter, Maximilien Kintz, Jens Drawehn. Vitrai: Applying Explainable ai in the Real World. In Kohei Arai, editor, Intelligent Systems and Applications, pages 11–23, Cham, 2022. Springer International Publishing

[24] Holzinger A, Plass M, Kickmeier-Rust M, Holzinger K, Crişan GC, Pintea C-M, Palade V (2019). Interactive Machine Learning: Experimental Evidence for the Human in the Algorithmic Loop. Appl Intell.

[25] Gabriele Ciravegna, Pietro Barbiero, Francesco Giannini, Marco Gori, Pietro Lió, Marco Maggini, Stefano Melacci (2021) Logic Explained Networks. arXiv preprint arXiv:2108.05149,

[26] Luca Veyrin-Forrer, Ataollah Kamal, Stefan Duffner, Marc Plantevit, and Céline Robardet (2022) On gnn explainability with activation rules. Data Mining and Knowledge Discovery, pages 1–35,

[27] Paul Tarau (2022) A gaze into the internal logic of graph neural networks, with logic. arXiv preprint arXiv:2208.03093,

[28] LucieCharlotte Magister, Dmitry Kazhdan, Vikash Singh, and Pietro Liò (2021) Gcexplainer: Human-In-The-Loop Concept-Based Explanations for Graph Neural Networks. arXiv preprint arXiv:2107.11889,

[29] Han Xuanyuan, Pietro Barbiero, Dobrik Georgiev, LucieCharlotte Magister, Pietro Lió (2022) Global concept-based interpretability for graph neural networks via neuron analysis. arXiv preprint arXiv:2208.10609,

[30] LucieCharlotte Magister, Pietro Barbiero, Dmitry Kazhdan, Federico Siciliano, Gabriele Ciravegna, Fabrizio Silvestri, Pietro Liò, Mateja Jamnik (2022) Encoding Concepts in Graph Neural Networks. arXiv e-prints, pages arXiv–2207,

[31] Steve Azzolin, Antonio Longa, Pietro Barbiero, Pietro Liò, Andrea Passerini (2022) Global explainability of gnns via logic combination of learned concepts. arXiv preprint arXiv:2210.07147

[32] Finale Doshi-Velez , Been Kim (2017) Towards a Rigorous Science of Interpretable Machine Learning. arXiv preprint arXiv:1702.08608

[33] Anna Hedström, Leander Weber, Dilyara Bareeva, Franz Motzkus, Wojciech Samek, Sebastian Lapuschkin, Marina M-C Höhne (2022) Quantus: An Explainable ai Toolkit for Responsible Evaluation of Neural Network Explanations. arXiv preprint arXiv:2202.06861,

[34] Hudec M, Minarikova E, Mesiar R, Saranti A, Holzinger A (2021). Classification by Ordinal Sums of Conjunctive and Disjunctive Functions for Explainable ai and Interpretable Machine Learning Solutions. Knowledge Based Systems.

[35] Schmid U, Finzel B (2020). Mutual Explanations for Cooperative Decision Making in Medicine. KI-Künstliche Intelligenz.

[36] Bruckert S, Finzel B, Schmid U (2020). The Next Generation of Medical Decision Support: A Roadmap Toward Transparent Expert Companions. Frontiers in artificial intelligence.

[37] Johannes Rabold, Hannah Deininger, Michael Siebers, Ute Schmid. Enriching Visual with Verbal Explanations for Relational Concepts - Combining LIME with Aleph (2019) In Peggy Cellier and Kurt Driessens, editors, Machine Learning and Knowledge Discovery in Databases - International Workshops of ECML PKDD 2019, Würzburg, Germany, September 16-20, 2019, Proceedings, Part I, volume 1167 of Communications in Computer and Information Science, pages 180–192. Springer,

[38] Andrew Cropper, Sebastijan Dumančić, StephenH Muggleton (2021) Turning 30: New Ideas in Inductive Logic Programming. In Proceedings of the Twenty-Ninth International Conference on International Joint Conferences on Artificial Intelligence, pages 4833–4839

[39] Muggleton SH, Schmid U, Zeller C, Tamaddoni-Nezhad A, Besold T (2018). Ultra-Strong Machine Learning: Comprehensibility of Programs Learned With ilp. Mach Learn.

[40] Ute Schmid, Christina Zeller, Tarek Besold, Alireza Tamaddoni-Nezhad, Stephen Muggleton (2016) How Does Predicate Invention Affect Human Comprehensibility? In International Conference on Inductive Logic Programming, pages 52–67. Springer,

[41] Frank P (1986). Expertensysteme. Inform Spektrum.

[42] Dash T, Srinivasan A, Vig L (2021). Incorporating Symbolic Domain Knowledge Into Graph Neural Networks. Mach Learn.

[43] Luc DeRaedt, Sebastijan Dumančić, Robin Manhaeve, and Giuseppe Marra (2020) From Statistical Relational to Neuro-Symbolic Artificial Intelligence. arXiv preprint arXiv:2003.08316

[44] Bettina Finzel, DavidE Tafler, Stephan Scheele, Ute Schmid (2021) Explanation as a Process: User-Centric Construction of Multi-Level and Multi-Modal Explanations. In German Conference on Artificial Intelligence (Künstliche Intelligenz), pages 80–94. Springer,

[45] Bettina Finzel, DavidElias Tafler, AnnaMagdalena Thaler, and Ute Schmid (2021) Multimodal Explanations for User-centric Medical Decision Support Systems. In ThomasE. Doyle, Aisling Kelliher, Reza Samavi, Barbara Barry, StevenJ. Yule, Sarah Parker, Michael Noseworthy, and Qian Yang, editors, Proceedings of the AAAI 2021, volume 3068 of CEUR Workshop Proceedings,

[46] Honghua Dong, Jiayuan Mao, Tian Lin, Chong Wang, Lihong Li, Denny Zhou (2019) Neural Logic Machines. arXiv preprint arXiv:1904.11694,

[47] Kexin Yi, Jiajun Wu, Chuang Gan, Antonio Torralba, Pushmeet Kohli, JoshuaB Tenenbaum (2018) Neural-Symbolic Vqa: Disentangling Reasoning From Vision and Language Understanding. arXiv preprint arXiv:1810.02338,

[48] Manhaeve R, Dumančić S, Kimmig A, Demeester T, DeRaedt L (2021). Neural Probabilistic Logic Programming in Deepproblog. Artif Intell.

[49] Hikaru Shindo, DevendraSingh Dhami, Kristian Kersting (2021) Neuro-Symbolic Forward Reasoning. arXiv preprint arXiv:2110.09383,

[50] Jiayuan Mao, Chuang Gan, Pushmeet Kohli, JoshuaB Tenenbaum, and Jiajun Wu (2019) The Neuro-Symbolic Concept Learner: Interpreting Scenes, Words, and Sentences From Natural Supervision. arXiv preprint arXiv:1904.12584,

[51] Yunchao Liu, Zheng Wu (2019) Learning to Describe Scenes With Programs. In International Conference on Learning Representations, pages 00–00,

[52] Chi Han, Jiayuan Mao, Chuang Gan, JoshuaB Tenenbaum, and Jiajun Wu (2020) Visual Concept-Metaconcept Learning. arXiv preprint arXiv:2002.01464

[53] Šourek G, Železnỳ F, Kuželka O (2021). Beyond Graph Neural Networks With Lifted Relational Neural Networks. Mach Learn.

[54] Mueller H, Holzinger A (2021). Kandinsky Patterns. Artificial intelligence.

[55] Birgit Pohn, Michaela Kargl, Robert Reihs, Andreas Holzinger, Kurt Zatloukal, Heimo Müller (2019) Towards a Deeper Understanding of How a Pathologist Makes a Diagnosis: Visualization of the Diagnostic Process in Histopathology. In IEEE Symposium on Computers and Communications (ISCC 2019), pages 1081–1086. IEEE,

[56] Andreas Holzinger, Bernd Malle, and Nicola Giuliani (2014) On Graph Extraction From Image Data. In International Conference on Brain Informatics and Health, pages 552–563. Springer,

[57] Graham Simon, QuocDang Vu, Shan EAhmed Raza, Ayesha Azam, YeeWah Tsang, JinTae Kwak, Nasir Rajpoot (2019) Hover-net: Simultaneous Segmentation and Classification of Nuclei in Multi-Tissue Histology Images. Medical Image Analysis 58:101563 10.1016/j.media.2019.101563 31561183

[58] Pushpak Pati, Guillaume Jaume, LaurenAlisha Fernandes, Antonio Foncubierta-Rodríguez, Florinda Feroce, AnnaMaria Anniciello, Giosue Scognamiglio, Nadia Brancati, Daniel Riccio, Maurizio DiBonito, etal (2020) Hact-net: A Hierarchical Cell-To-Tissue Graph Neural Network for Histopathological Image Classification. In Uncertainty for Safe Utilization of Machine Learning in Medical Imaging, and Graphs in Biomedical Image Analysis, pages 208–219. Springer,

[59] MichaelM Bronstein, Joan Bruna, Taco Cohen, and Petar Veličković (2021) Geometric Deep Learning: Grids, Groups, Graphs, Geodesics, and Gauges. arXiv preprint arXiv:2104.13478,

[60] ThomasN Kipf ,Max Welling (2016) Semi-supervised Classification With Graph Convolutional Networks. arXiv preprint arXiv:1609.02907,

[61] Holzinger A, Langs G, Denk H, Zatloukal K, Müller H (2019). Causability and Explainability of Artificial Intelligence in Medicine. Wiley Interdisciplinary Reviews: Data Mining and Knowledge Discovery.

[62] MacKay DJC, MacKay DJC (2003). Information Theory.

[63] Dongsheng Luo, Wei Cheng, Dongkuan Xu, Wenchao Yu, BoZong, Haifeng Chen, and Xiang Zhang (2020) Parameterized Explainer for Graph Neural Network. arXiv preprint arXiv:2011.04573,

[64] Bach S, Binder A, Montavon G, Klauschen F, Müller K-R, Samek W (2015). On Pixel-Wise Explanations for Non-linear Classifier Decisions by Layer-Wise Relevance Propagation. PLoS ONE.

[65] Zhaoning Yu, Hongyang Gao (2022) Motifexplainer: A Motif-Based Graph Neural Network Explainer. arXiv preprint arXiv:2202.00519

[66] MarcoTulio Ribeiro, Sameer Singh, Carlos Guestrin (2016) Why Should I Trust You?: Explaining the Predictions of Any Classifier. In 22nd ACM SIGKDD International Conference on Knowledge Discovery and Data Mining (KDD 2016), pages 1135–1144. ACM,

[67] Anna Saranti, Behnam Taraghi, Martin Ebner, Andreas Holzinger (2019) Insights Into Learning Competence Through Probabilistic Graphical Models. In International cross-domain conference for machine learning and knowledge extraction, pages 250–271. Springer

[68] Daphne Koller, Nir Friedman (2009) Probabilistic Graphical Models: Principles and Techniques. MIT press,

[69] Hao Yuan, Jiliang Tang, Xia Hu, Shuiwang Ji (2020) Xgnn: Towards Model-Level Explanations of Graph Neural Networks. In Proceedings of the 26th ACM SIGKDD International Conference on Knowledge Discovery & Data Mining, pages 430–438,

[70] Ashwin Srinivasan. The Aleph Manual. http://www.cs.ox.ac.uk/activities/machinelearning/Aleph/

